# Supplementary material for: High-fidelity simulation versus case-based tutorial sessions for teaching pharmacology: Convergent mixed methods research investigating undergraduate medical students’ performance and perception
Source: PLoS One. 2024 Aug 16;19(8):e0302609. doi: 10.1371/journal.pone.0302609 (PMC11329139; doi:10.1371/journal.pone.0302609)
Supplement: S1 Data — (DOCX) [file pone.0302609.s001.docx]

| Theme | Category | Exemplar |
| --- | --- | --- |
| Variation of experience | Interactions | 3/S: “It’s more interactive and realistic.”  7/S: “The simulation allowed us to implement what we learned in class as clinical cases that we will encounter and made the session very interactive and interesting.”  24/S: “Interacting with patients helped me to understand how quickly decisions must be taken.”  18/T: “The inter-group discussions that were later followed by discussing our answers with the other groups and Dr. Rajan was very effective and helped me assess my understanding better.”  36/T: “I found the simulation more interactive and engaging. The information was immediately instilled in my mind during the simulation; however, the more traditional method of tutorials is not as engaging and although group work is involved, it feels more like a lecture.” |
|  | Movement | 22/S: “We learned how to administer the drugs in real-life situations, along with important details to make sure are done before administration like the mode of administration for every drug as well as the preservation of the drug.”  22/T: “I think this is more relevant at later stages of our learning, but the tutorials could be more student-guided if it was in a PBL/flipped learning style where there is a long case analyzed by students and then the findings could be presented to colleagues and the professor.”  9/T: “Maybe we could have some pictures or videos to watch and then solve the cases in the videos.” |
| Reinforcing learning | Skills | 12/S: “Seeing it practically applied to patients helps the thinking process.”  16/S: “It helped me apply the information to a real-life scenario and how to act and make decisions fast in life-threatening situations.”  31/S: “Answering questions in a clinical setting is different. through this session, it improved my confidence when it comes to interacting with patients and thinking of answers on the spot.”  45/S: “I was able to put my theoretical knowledge into practice by understanding the cases presented.”  9/T: “It helped us use the drugs we learned to treat patients.” |
|  | Knowledge | 2/S: “Yes, I found it very helpful even though I forgot to revise but I feel like it will help me retain the information better.”  10/S: “It better helped me understand the lesson since it is more practical, and I find it easier to remember things by seeing them and pointing them out myself rather than just solving questions in class.”  21/S: “Watching drug administration in a clinical setting is more helpful when it comes to learning.”  24/S: “Interacting with patients helped me to understand how quickly decisions must be taken and why certain routes will be preferred more than others. I also realized that the intake of drugs is more convenient through certain routes than others.”  5/T: “Since the cases uses real life cases with medication names, it incorporates what we studied and makes it stick in our head.”  3/T: “It used applied concepts which made me better understand the drugs and routes of administration.”  34/T: “I preferred the simulation as I didn’t learn all the cases properly.” |
|  | Attitudes | 37/S: “It gave me an insight to what actually happens in medical practice.”  12/T: “Tutorial cases reenforced the lectures main points/objectives.”  26/T: “it helped support but in my point of view that when we had simulation the information where better and easier understood since its more interactive.”  41/T: “Simulation helps to reinforce the concept more.” |
| Level of realism | Fidelity | 4/S: “I enjoyed putting the theory into practice. Seeing what we learned in class applied in a scenario that mimics a real-life situation encouraged me to participate and actively learn.”  9/S: “It reinforced the important concepts and helped us experience real-life scenarios that would help us intervene and reach a faster analysis in real-life situations when patients present with similar conditions.”  8/S: “We saw applications of everything we did in class which was very helpful because it was in a clinical setting.”  13/T: “Yes it gave me a real-life outlook of what I learnt.”  39/T: “It gives a possible real-life scenario to apply what we learnt and integrates other courses as well since we are taking a look at the bigger picture.” |
|  | Immersiveness | 23/S: “It helps put the relatively calm theory into the stressful hospital environment [more stressful but more realistic].”  23/S: “I find this more stressful, but better here than a shock in the hospital with the real thing. this helps put things in perspective and prepare us for the real thing.”  14/T: “We learned how to think like a pharmacist.” |
